# The landscape of dementia research in Brazil from 2010 to 2021: a Scopus-based bibliometric study

**DOI:** 10.1055/s-0044-1792095

**Published:** 2024-11-29

**Authors:** Ari Alex Ramos, Laiss Bertola, Fabiana Araújo Figueiredo da Mata, Andrew Christopher Claro Miguel, Haliton Alves de Oliveira Junior, Cleusa Pinheiro Ferri

**Affiliations:** 1 Hospital Alemão Oswaldo Cruz, Sustentabilidade e Responsabilidade Social, São Paulo SP, Brazil.; 2 Universidade Federal de São Paulo, Escola Paulista de Medicina, Departamento de Psiquiatria, São Paulo SP, Brazil.

**Keywords:** Scientific and Technical Publications, Bibliometrics, Data Science, Cognitive Aging, Dementia, Publicações Científicas e Técnicas, Bibliometria, Ciência de Dados, Envelhecimento Cognitivo, Demência

## Abstract

**Background** Several studies have sought to investigate the trajectory of scholarly publications on dementia. Yet, there has been limited attention to contributions from Latin America.

**Objective** To provide a comprehensive overview of the literature output on dementia in Brazil.

**Methods** We conducted a Scopus-based literature search (2010–2021) for publications by authors affiliated with Brazil.

**Results** Out of 5,534 reports, 2,528 met the inclusion criteria. The annual growth rate of publications on dementia (9.9%, SD = 15.5) closely paralleled that of general health-related literature (6.7%, SD = 4.9). Most publications were categorized into the areas of diagnosis (33.4%) and disease mechanisms, origins, and models (32.7%). Epidemiological studies (4%), clinical trials (1%), and economic analyses (0.3%) are scarce. Based on the first affiliation of Brazil-affiliated authors, 89.3% of dementia output stemmed from Southeast (68.4%) and South (20.9%) of Brazil. Nonetheless, the state of São Paulo alone accounted for 41.1%, contributing to 60.1% of the Southeast. First and second authorships were predominantly held by female researchers, whereas male researchers occupied most of the second-to-last and last authorships. Overall, 1,812 (71.7%) were published in 346 foreign journals and 716 (28.3%) in 43 Brazilian journals. Notably, nearly half of the reports published in Brazil are concentrated in two journals: Dementia e Neuropsychologia (31.4%) and Arquivos de Neuro-Psiquiatria (15.2%).

**Conclusion** There is a pressing need for more studies in dementia epidemiology and economic cost, in addition to more research across all Brazilian regions.

## Resumo

**Antecedentes** Diversos estudos têm buscado investigar a trajetória das publicações acadêmicas sobre demência. Entretanto, as contribuições da América Latina recebem pouca atenção.

**Objetivo** Prover uma revisão bibliométrica abrangente da produção científica sobre a demência no Brasil.

**Métodos** A busca da literatura foi conduzida no Scopus (2010–2021) e englobou as publicações de autores afiliados ao Brasil.

**Resultados** Das 5.534 publicações encontradas, 2.528 preencheram os critérios de inclusão. A taxa de crescimento anual das publicações sobre demência (9,9%, DP = 15,5) é semelhante à da literatura geral em saúde (6,7%, DP = 4,9). A maioria das publicações foi categorizada nas áreas de diagnóstico (33,4%) e mecanismos, origens e modelos da demência (32,7%). Estudos epidemiológicos (4%), ensaios clínicos (1%) e análises econômicas (0,3%) são escassos. Aproximadamente 89% das primeiras afiliações provieram do Sudeste (68,4%) e do Sul (20,9%). Entretanto, o estado de São Paulo, sozinho, respondeu por 41,1%, contribuindo com 60,1% de toda a região Sudeste. A primeira e a segunda autorias foram ocupadas principalmente por pesquisadoras do sexo feminino; em contraste, os pesquisadores do sexo masculino ocuparam a maioria da penúltima e última autorias. No total, 1.812 (71,7%) documentos foram publicados em 346 periódicos estrangeiros e 716 (28,3%) em 43 periódicos brasileiros. Notadamente, quase metade das publicações no Brasil está concentrada em dois periódicos científicos: Dementia e Neuropsychologia (31,4%) e Arquivos de Neuro-Psiquiatria (15,2%).

**Conclusão** Conclui-se que há a necessidade de mais estudos epidemiológicos e avaliações econômicas sobre o custo da demência, além de mais pesquisas sobre a demência nas cinco regiões do Brasil.

## INTRODUCTION

Over the past two decades, several studies have been conducted to assess the growth of scholarly publications on dementia. In a previous study, Theander and Gustafson 1 investigated the dementia-related research trajectory based on literature indexed in Medline, spanning 35 years (1974–2009). The findings showed that the number of dementia-related publications, relative to the total number of publications indexed in Medline, surged from 0.17% (467/272,502) in 1970 to 0.78% (3,888/486,732) in 2000, indicating that these studies exhibited a more accelerated growth rate compared to the medical literature. More recently, Dong et al. 2 conducted an extensive bibliometric review (1988–2017) on Alzheimer's disease (AD), including 181,116 publications. The primary outcomes revealed that studies in basic research (i.e., neurobiological mechanisms of the syndrome) represent 30.9% of the global literature on AD. Notably, the United States, England, Germany, Japan, and Italy accounted for 67.9% of global output, underscoring that dementia-related research is predominantly conducted by high-income countries. 2

Another bibliometric study 3 focused on the Brazilian scientific production (indexed in Scopus) regarding AD before (1974–2005) and after (2006–2019) the implementation of the National Agenda for Health Research Priorities. The results indicated a substantial increase in the number of original articles and literature reviews on the topic during the second period analyzed (2006–2019). The authors suggest that adopting the health research priority policy played a role in fostering more studies and led to an increased number of publications on AD from Brazil-affiliated researchers. In the current bibliometric review, we expand on this previous study by providing further insightful findings on the scientific output of research stemming from Brazil, covering not only AD but also the most common subtypes of dementia ( Box 1 ).

**Box 1:** Types of dementia included in the bibliometric analysis

Type of dementia
Alzheimer's disease
Frontotemporal dementia
Huntington disease
Lewy body dementia
Parkinson's disease dementia
Pick's disease
Primary progressive aphasia
Primary progressive nonfluent aphasia
Vascular dementia

Past bibliometric analyses 2 4 5 have also indicated that literature on dementia is primarily concerned with diagnosis (e.g., neuropsychological assessment and magnetic resonance imaging [MRI]) and the neurobiological mechanisms of the syndrome (e.g., oxidative stress, beta-amyloid protein deposition, neurofibrillary tangles). In contrast, there is a global scarcity of studies on the economic impact of dementia. Investigations into diagnosis and neurobiological mechanisms are pivotal from a scientific standpoint and essential for enhancing clinical practice. Despite this, other research areas should be prioritized when considering the implications of treating and managing dementia. In this regard, the World Health Organization (WHO) has formulated a strategic research plan encompassing the entire spectrum of dementia syndrome. This initiative is encapsulated in a recent report, 6 which outlines six specific research areas:

- Dementia epidemiology and economic cost;
- Dementia disease mechanisms, origins, and models;
- Dementia diagnosis;
- Drug development and clinical trials for dementia;
- Dementia care; and
- Dementia risk reduction. 7

The mapping of research areas stemmed from similar strategies previously adopted by the WHO in addressing infectious diseases such as malaria and other epidemics. Hence, prioritizing research areas, specifically for dementia, marks the first initiative of this nature in the context of noncommunicable chronic diseases.

In light of the pressing need for more studies on dementia in low- and middle-income countries, along with the necessity of a more accurate understanding of the scientific production on dementia in Brazil, the objective of this bibliometric review was to document and measure the contribution to dementia research among researchers affiliated with Brazil. Moreover, the specific objectives of this review were as follows:

- To contrast the average annual growth rate of dementia publications versus general health publications;
- To classify each analyzed publication according to the research areas recently defined by the WHO;
- To compare the scientific production among the five Brazilian geographical regions;
- To characterize the authorship roles of researchers affiliated with Brazil;
- To identify the leading Brazilian and foreign journals of publication and the top 10 funding agencies cited in the analyzed publications;
- To investigate the contribution of female researchers, compared to male researchers, in the study of dementia, in line with the spirit of gender equality in scientific research.

## METHODS

The literature search was conducted in the Scopus database on September 28, 2022, covering publications indexed between January 2010 and December 2021. To streamline the screening process through titles and abstracts, we imported the records identified into the JBI Summary online platform ( https://jbi-global-wiki.refined.site/space/SKB ) – a tool developed for conducting literature reviews. Two researchers independently examined the titles and abstracts of all records to determine initial eligibility. Subsequently, the full version of potentially eligible reports was retrieved, and the same authors completed the assessment. Discrepancies regarding eligibility were initially resolved through consensus between both researchers and, when necessary, another senior researcher in dementia was involved.

To elucidate the potential increase in publications on dementia in the context of Brazilian scientific production within the broader health landscape, we conducted a supplementary literature review that aimed to ascertain the total volume of Scopus-indexed publications authored by researchers affiliated with Brazil during the specified period (2010–2021). To refine the literature search and focus exclusively on health-related literature, we selected the following areas within the filter “subject area”: pharmacology, medicine, psychology, and health professions.

Additionally, we marked “Brazil” within the country/territory filter to retrieve publications that featured authors affiliated with Brazil, irrespective of their authorship positions. This methodological approach enabled us to compute the mean annual growth rate of health-related publications versus dementia-related reports. Furthermore, it allowed testing potential statistically significant differences between these two growth rates through an independent two-sample *t* -test. The **Supplementary Material** (available at https://www.arquivosdeneuropsiquiatria.org/wp-content/uploads/2024/09/ANP-2024.0112-Supplementary-Material.docx ) shows the search strategy used in the present bibliometric analysis, and Box 1 specifies the prevailing types of aging-associated dementia analyzed in the current study.

### Eligibility criteria

We included original articles, literature reviews, letters to the editor and editorials published between 2010 and 2021. The language of publication – English, Portuguese, or Spanish – was an additional criterion adopted in this bibliometric review.

Regarding basic research (e.g., in molecular and cellular biology) with or without animal models, we considered for inclusion only those studies that articulated a direct association or implication with the types of dementia listed in Box 1 . For instance, we included research utilizing beta-amyloid-induced Alzheimer's in animal models while excluding studies investigating the neurobiological activity of acetylcholinesterase devoid of a direct association with the targeted dementia conditions.

These criteria allowed a comprehensive analysis of a considerable number of publications focused on the primary dementia conditions that are common in pathological aging. Therefore, we excluded publications involving exclusively samples of dementia-affected individuals due to chronic affective disorders (e.g., bipolar disorder), Down syndrome, HIV encephalopathy, spinocerebellar ataxias (e.g., Machado-Joseph disease), prion diseases (e.g., Creutzfeldt-Jakob disease), and rare genetic abnormalities (e.g., Kluver-Bucy syndrome).

### Research areas in dementia according to categories proposed by the WHO

Due to the objectives and specificities of this bibliometric review, we expanded the proposed six research areas in dementia outlined by WHO (epidemiology and economic cost, disease mechanisms, origins, and models, diagnosis, drug development and clinical trials, dementia care, and risk reduction), 7 which involved disaggregating drug development and clinical trials and categorizing epidemiology and economic cost as separate areas.

This adjustment provided a more nuanced and comprehensive overview of the dementia-related literature authored by researchers with academic affiliations in Brazil. Furthermore, within the drug development area, we grouped publications investigating pharmacological properties of plants (e.g., potential anticholinesterase effect of green tea) or compounds with potential therapeutic activity (e.g., alkaloids), drugs under development, drugs already in use for treatment (e.g., rivastigmine and donepezil) or with potential therapeutic effects (e.g., lithium carbonate), in addition to studies focusing on the general pharmacological treatment of dementia. Note that the clinical trials included in this bibliometric review were not limited to drug development trials (as proposed initially by WHO) but encompassed any research employing the methods that characterize a clinical trial – this was driven by the interest in determining the number of dementia-related clinical trials published in the last decade.

In the area of diagnosis, we considered publications on biomarkers (measurement of beta-amyloid and tau proteins), neuroimaging techniques and brain mapping (computed tomography [CT], MRI, and electroencephalogram [EEG]), neuropsychological/cognitive assessment, and other relevant tools for the clinical diagnosis of dementia. In the area of disease mechanisms, origins, and models, we included publications investigating the neurobiology of dementia (e.g., genetic mutation, neuroinflammation, oxidative stress, mitochondrial dysfunction) with or without the use of dementia syndrome models (e.g., animal ex-vivo, or computational model).

### Data extraction and coding

The data extracted and coded for each publication included in this review were based on the following characteristics:

- Categorization of each publication included according to the respective dementia research area defined by WHO;
- Name and sex of the first, second, second-to-last, and/or last author, with the goal of identifying and quantifying the leading role in authorship of researchers affiliated with Brazil;
- The Brazilian geographical regions linked to each publication, based on the first affiliation in Brazil of the first, second, second-to-last, and/or last author;
- Determination of the journal of publication as Brazilian or non-Brazilian; and
- Identification of research funding sources when available.

We developed specific syntaxes in the R (R Foundation for Statistical Computing, Vienna, Austria) software, 8 with the RStudio interface, 9 for all stages of data extraction and classification of relevant information. For instance, key terms and expressions created by the authors and derived from the WHO report 6 were included in coded functions in R to allow the preliminary categorization of each publication within the respective dementia research area. Subsequently, two researchers carefully analyzed the automatically generated results to check and ensure data consistency and resolve any conflict, such as publications categorized in more than one area. In instances of conflict, the overarching objective of the study determined the research area (e.g., dementia care, epidemiology, diagnosis, economic cost).

The attribution of sex for authors with leading positions in authorships and affiliation with Brazil was ascertained based on the authors' names and, for a small proportion, through physical appearance, as found in photos on Google Scholar, LinkedIn, ResearchGate, and other social networks. Note that similar approaches have been adopted in previous bibliometric analyses. 10 Two researchers cross-validated data generated from implemented R syntaxes for sex attribution.

Following the National Institute of Health (NIH), 11 in the current bibliometric review we employed the term “sex” as a biological variable that categorizes individuals as male or female. To classify the journal of publication as either Brazilian or non-Brazilian, we first downloaded files containing all journals listed by the SCImago Journal Rank (SJR) as originating from Brazil ( https://www.scimagojr.com/journalrank.php?country=BR ) and indexed in Scopus in the period between 2010 and 2021. Using R-developed syntaxes, journals not cataloged in this initial listing were deemed non-Brazilian. Before carrying out the analyses, we manually reviewed the list of journals generated by SJR to rectify any inconsistencies.

### Ethical approval

The present study is part of the ReNaDe Project (National Report on Dementia in Brazil). Ethical approval was granted according to CAAE n°. 58125222.6.0000.0070.

## RESULTS

**Figure 1:**
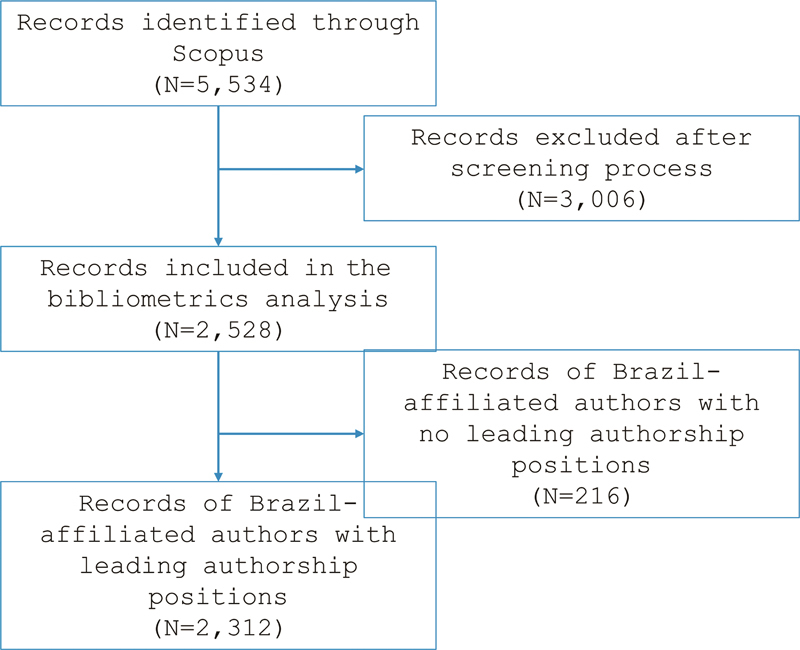
Flow diagram of study selection.

Figure 1 illustrates the flow diagram of study selection. Out of 5,534 publications found in Scopus, 2,528 met the inclusion criteria. The initial analyses yielded the following noteworthy bibliometric data: 10,821 authors, of which 7,343 held both leading positions in authorship and affiliations with Brazil; 37.6% of the included publications featured international coauthorship; 45 publications contained only one author; 95.1% of the analyzed documents were published in English and 4.5% in Portuguese; and the average citation per document in Scopus was calculated at 29.8.

In the **Supplementary Material** , **Figure S1** illustrates the year-wise distribution of the 2,528 documents analyzed, whereas **Figure S2** displays the trajectory of dementia-related production compared to general health output. The number of publications surged from 118 in 2010 to 301 in 2021, with an estimated average annual growth rate of 9.9% (standard deviation [SD] = 15.5). Meanwhile, the number of general health publications increased from 16,449 in 2010 to 33,259 in 2021, with an estimated average annual growth rate of 6.7% (SD = 4.9). Nonetheless, we did not find statistically significant differences between the two growth rates (t _12_ = 0.65, *p* = 0.53). Indeed, the proportion of publications on dementia compared to the total number of health-related publications ranged from 0.68 to 1.02%, with no clear trend of increase or decrease during the period analyzed (2010–2021).

**Table S1** in the **Supplementary Material** describes the types of publications identified. Overall, there was a predominance of original articles (79.0%), followed by literature reviews (17.4%), letters to the editor (2.1%), and editorials (1.4%). However, the proportion of original articles declined from 83.9 in 2010 to 73.8% in 2021, while literature reviews increased from 14.4 in 2010 to 24.2% in 2021. We did not observe a substantial increase when comparing the proportions of letters to the editor and editorials in 2010 and 2021.

### Research areas in dementia according to categories proposed by the WHO

**Figure 2:**
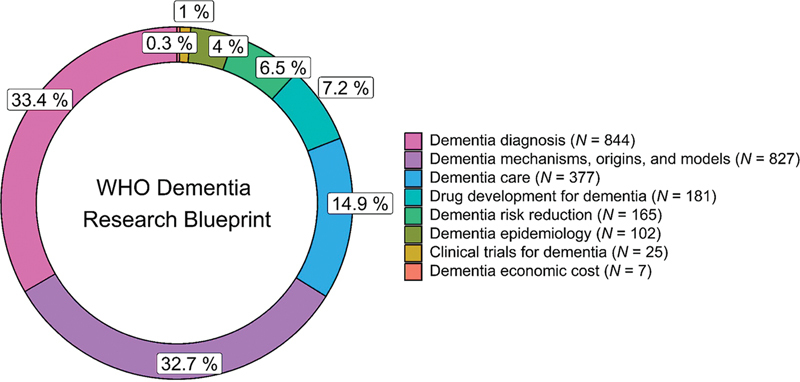
Distribution of the publications analyzed by research area in dementia according to the World Health Organization (WHO).

**Figure 3:**
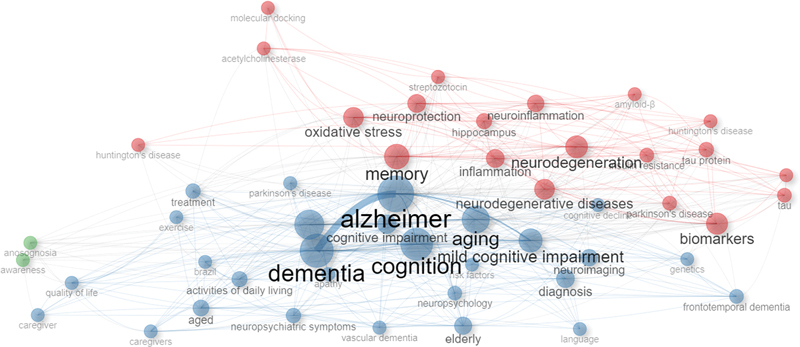
Thematic map based on the 50 most frequent authors' keywords.

Each publication included in this review was categorized according to the dementia research areas defined by the WHO. As illustrated in Figure 2 , over 65% of the analyzed publications focused on either diagnosis (33.4%) or mechanisms, origins, and models (32.7%). Dementia care (14.9%), drug development (7.2%), risk reduction (6.5%), and epidemiology (4%) appeared in sequence. Finally, there were notably low percentages of publications related to clinical trials (1%) and economic costs (0.3%). The thematic map in Figure 3 was drawn from the 50 most frequent author's keywords and illustrates the concentration of dementia research on diagnosis and disease mechanisms, origins, and models.

When comparing the number of publications in 2010 ( *N* = 118) and 2021 ( *N* = 301), **Table S2** ( **Supplementary Material** ) shows a surge in publications across all areas. Nevertheless, there was a drop in the proportion of publications on diagnosis, declining from 44.1 to 26.9%. In contrast, there was an upward trend in the proportion of publications addressing disease mechanisms, origins, and models, rising from 23.7 to 31.6%. Proportionally, there were more studies on risk reduction in 2010 (9.3%) compared to 2021 (7.6%). For drug development and epidemiology, percentages were below 2% in 2010, increasing to 7% in 2021. There were no publications of clinical trials or economic costs in 2010; after 11 years, the percentage of published reports in these two areas remained notably low (2 and 0.3%, respectively).

In recent years, publications on dementia by Brazil-affiliated authors have increasingly focused on studies involving disease mechanisms, origins, and models (i.e., basic research). This shift is evidenced in **Figure S3** , which illustrates the publication trend for the three areas of research in dementia with the highest number of publications between 2010 and 2021. A visual inspection of **Figure S3** reveals that the area of diagnosis peaked in 2011 ( *N* = 81) and relatively stabilized from 2018 to 2021. However, the number of publications on disease mechanisms, origins, and models of dementia surpassed those on diagnosis after 2016. Mechanisms, origins, and models of dementia exhibited a relatively continuous growth trajectory since 2010 ( *N* = 28), reaching its peak in 2020 ( *N* = 95). In relation to dementia care, there was a peak in scientific output in 2018 ( *N* = 41), followed by a steady increase in publications from 2019 to 2021.

### Geographical regions of Brazil

**Figure 4:**
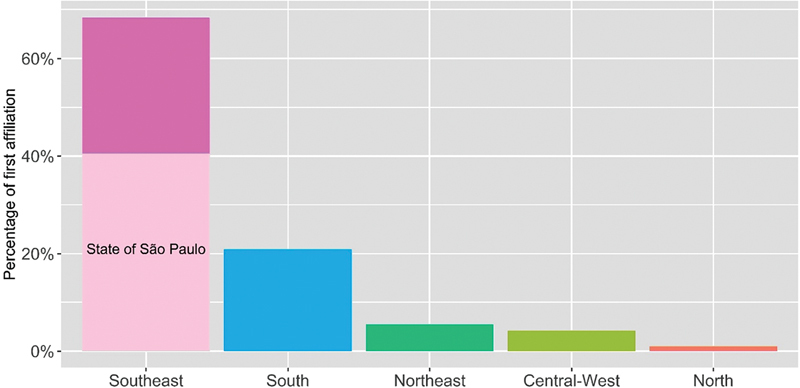
Percentage of first affiliations by geographical region. The primary affiliation in Brazil of the first, second, second-to-last, and/or last author was considered to calculate the percentage of affiliations.

The analysis of the geographic regions was limited to the first affiliation in Brazil of the first, second, second-to-last and/or last authors, encompassing a total of 7,343 authors and 2,312 publications. It is important to note that 216 publications lacked authors affiliated with Brazil in these leading positions. Figure 4 illustrates that 68.4 and 20.9% of these authors reported research institutions from the Southeast and South, respectively, as their first (primary) affiliation in Brazil. Hence, over 90% of the first affiliations analyzed here originate from those two regions, followed by Northeast (5.5%), Central-West (4.2%), and North (1.0%). Notably, while the Southeast region comprises 68.4% of first affiliations, the state of São Paulo itself constitutes 41.1%, contributing to 60.1% of the whole Southeast Region. Thereby, the percentage attributed to this state surpasses the combined percentages of all other four regions (31.6%).

**Table S3** ( **Supplementary Material** ) displays the distribution of the affiliations under analysis in 2010 ( *N* = 362) and 2021 ( *N* = 850) by geographic region. Except for the North, there has been a substantial increase in the number of first affiliations in the remaining four regions. In the Southeast, however, we noticed a decline in the proportion of first affiliations, dropping from 76.5 in 2010 to 64.4% in 2021. Conversely, the proportion of first affiliations in the Northeast rose from 1.4 in 2010 to 8.9% in 2021. The Southern and Central-Western regions witnessed a modest increase in the proportion of first affiliations (South: 20.4–23.8%; Central-West: 1.4–2.9%). As shown on **Figure S4** , there was an increasing number of affiliations during the period analyzed, particularly in the Southeast and South of Brazil.

### Leading roles in authorships

Out of the 2,528 publications analyzed, 91.5% ( *N* = 2,312) featured Brazil-affiliated authors in a leading position (first, second, second-to-last and/or last author). In the present bibliometric review, we were particularly interested in documenting the leading role of women in dementia publications. To this end, we initially determined the sex of the leading authors in each publication. **Figure S5** illustrates that, except for the period between 2011 (49.5%) and 2012 (47.7%), female protagonism in authorship consistently remained at 50% or higher in the subsequent years, reaching its peak in 2018 (59.8%).

**Figure 5:**
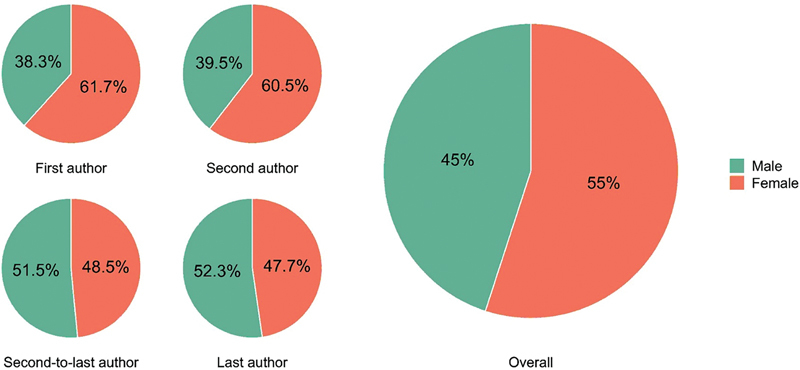
Proportion of female researchers with affiliation in Brazil.

If we combine the number of first, second, second-to-last and/or last authors affiliated with Brazil, women accounted for 55% of the protagonism. Figure 5 shows that female protagonism exceeded 60% in the first and second author positions. In contrast, there was a male protagonism in the second-to-last (51.5%) and last (52.3%) author positions. We also analyzed women's contribution to the research areas in dementia defined by WHO. Except for diagnosis (45%), women constituted over half of authors taking on leading authorship roles, with the two research areas with highest rates being dementia care (68%) and economic cost (61%).

### Top 10 journals of publication

Among the 2,528 documents analyzed, 1,812 (71.7%) were published in 346 foreign journals and 716 (28.3%) in 43 Brazilian journals. **Figure S6** in the **Supplementary Material** shows a singular instance, in 2011, where the ratio of publications in Brazilian journals (50.9%) marginally surpassed their international counterparts (49.1%).

**Figure 6:**
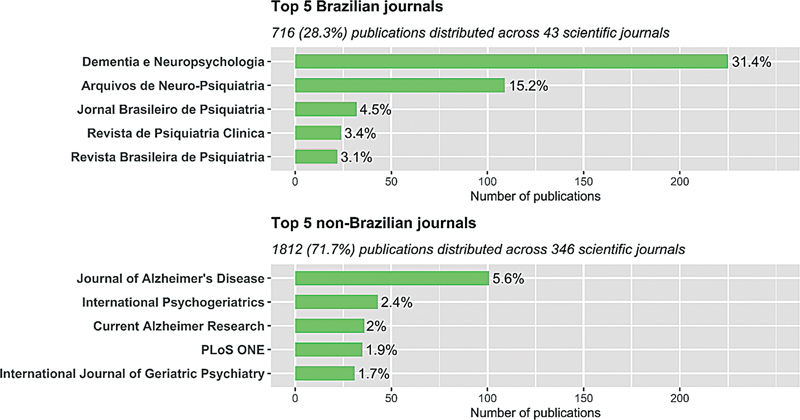
The top 10 journals for publishing dementia research conducted by authors affiliated with Brazil.

Overall, there has been an increase in the proportion of publications in foreign journals, reaching 80.1% in 2021. Figure 6 reveals that the journal Dementia & Neuropsychologia is the Brazilian journal with the highest number of publications on dementia, contributing to over 30% of all publications in Brazilian journals. Subsequently, the Arquivos de Neuro-Psiquiatria journal stands out, hosting more than 15% of the documents published. The Jornal Brasileiro de Psiquiatria (Brazilian Journal of Psychiatry), the Revista de Psiquiatria Clínica (Archives of Clinical Psychiatry), and the Revista Brasileira de Psiquiatria collectively account for 11% of the related publications in Brazil.

In contrast, there was no high percentage of publications concentrated in just one foreign journal. This reflects the dispersion of the 1,812 manuscripts published abroad, across a more extensive array of journals (346 foreign vs. 43 Brazilian). The top 5 foreign journals for authors affiliated with Brazil accounted for 13.6% of the publications included in this bibliometric review, with the Journal of Alzheimer's Disease standing out, at 5.6%. The other 4 top non-Brazilian journals (International Psychogeriatrics, Current Alzheimer Research, Plos ONE, and International Journal of Geriatric Psychiatry) jointly published 8% of the foreign publications.

### Funding agencies acknowledged in the publications analyzed

**Figure 7:**
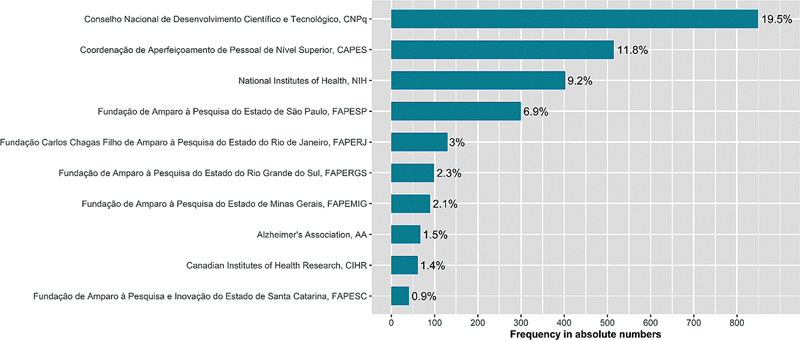
The top 10 funding agencies identified in the publications analyzed. **Notes:** The values indicated at the end of each horizontal bar correspond to the percentage of publications funded by the respective funding agency. To tally the funding agencies mentioned in the publications analyzed, information about funding and financial resources from institutions that are part of the National Institutes of Science and Technology Program (Programa Institutos Nacionais de Ciência e Tecnologia – INCTs) were grouped under the acronym CNPq. Similarly, under the umbrella term National Institutes of Health, we aggregated the present-day 27 institutes and research centers that comprise the United States' NIH ( https://www.nih.gov/institutes-nih/list-institutes-centers ).

In the analyses of research funding agencies, we included the financial resources reported by all authors from the 2,528 publications. Figure 7 lists the top 10 funding agencies (both national and international) cited in the publications included in this bibliometric review. More than ⅓ of the publications analyzed received financial support from the two main research funding agencies in Brazil, namely the National Council for Scientific and Technological Development (CNPq, 19.5%) and the Coordination for the Improvement of Higher Education Personnel (CAPES, 11.8%). The United States' NIH is the third-ranking research funding agency, acknowledged in 9.2% of the analyzed publications. This aligns with the results that show 37.6% of the included publications featured international collaborative authorship, including researchers affiliated with American institutions.

In line with the aforementioned outcome, that 41.1% of authors taking on leadership roles in authorships included research institutions from São Paulo as their principal affiliation in Brazil, nearly 7% of the publications reported receiving financial support from the São Paulo Research Foundation (FAPESP). In fact, CNPq, CAPES, and FAPESP stand out as Brazil's top 3 research funding agencies, cited individually or collectively in 38.2% of the publications analyzed. Furthermore, research support foundations from the States of Rio de Janeiro (FAPERJ), Rio Grande do Sul (FAPERGS), Minas Gerais (FAPEMIG), and Santa Catarina (FAPES) were listed, separately or collectively, in 8.3% of the publications. The Alzheimer's Association and the Canadian Institute of Health Research emerged as the two leading international funding agencies, mentioned individually or collectively in 3% of the analyzed publications.

## DISCUSSION

This bibliometric review aimed to map the scientific production, indexed in Scopus, on the core clinical types of aging-associated dementia published by authors affiliated with Brazil. The findings indicate that dementia-related output in Brazil presented an annual growth rate similar to that of overall health production between 2010 and 2021.

Most of the documents analyzed pertain to original articles (79.0%) and literature reviews (17.4%). Nevertheless, an examination of percentages of publications, specifically in 2010 and 2021, reveals a rise in the proportion of literature reviews at the expense of original studies. On the one hand, this result likely reflects the increasing demand for synthesizing data from empirical research through literature reviews and, on the other hand, potentially the fact that Brazilian researchers currently possess more excellent proficiency in the methods and techniques involved in systematic reviews and meta-analyses.

Although the number of letters to the editor and editorials has increased, the percentages of publications in these two modalities of scientific communication amount to only 2.1 and 1.4%, respectively. In the global research on AD, 4 the proportion of brief communications, including letters to the editor and editorials, declined from 1970 to 2014. In the current study, however, the volume of publications in these two modalities of scientific communication is distributed almost evenly across the analyzed period (2010–2021), indicating no substantial proportional growth from one year to the next.

To the best of our knowledge, this is the first bibliometric review that categorized the publications analyzed according to the dementia research areas recently defined by the WHO. 7 Notably, the results revealed that over 65% of the publications included were ranked in the areas of diagnosis (33.4%) or of mechanisms, origins, and models of dementia (32.7%). Remarkably, the number of publications in the latter area exceeded that of the former in more recent years, indicating that basic research on the pathophysiological mechanisms of dementia has gained increased prominence in publications by authors affiliated with Brazil. This trend aligns with findings from previous studies 2 4 5 that were not limited to authors affiliated with Brazil. For example, basic research with animal models in AD investigation has continuously increased since the 1990s, concurrently with a growing number of publications on potential genetic mechanisms associated with this syndrome. 5

The necessity of expanding the scope of research to cover all dementia scenarios, as postulated by the WHO, 6 becomes evident in the current bibliometric review, which shows a modest proportion of publications categorized in the areas of risk reduction (6.5%), drug development (7.2%), and epidemiology (4%). Furthermore, studies involving clinical trials (1%) and economic cost (0.3%) are even scarcer. In Brazil, the foreseeable number of people with dementia will triple by 2050. 12 Therefore, studies focused on prevention and economic cost are essential to reduce the prevalence and minimize the costs of dementia in the country. The need for more studies on prevention that inform the development of public policies is reaffirmed, given that more than ⅓ of dementia cases are related to modifiable risk factors, 13 more specifically, behaviors and lifestyle habits such as low education, hypertension, obesity, physical inactivity etc.

Approximately 68% of the first, second, second-to-last and/or last authors reported research institutions in the Southeast region as their first affiliation, which seems reasonable, considering that this region harbors the highest number of universities engaged in academic research. However, in subsequent analysis, we discovered that 41.1% of the first affiliations under examination are located in the state of São Paulo, that also concentrates 60.1% of the affiliations originating from research institutions in the Southeast. We speculate that this finding reflects two main factors: first, the high concentration of researchers and universities (both public and private) in this State; second, the data from this bibliometric review show that FAPESP was the third most cited research funding institution. This line of reasoning aligns with a prior analysis of Brazilian scientific output indexed in the Web of Science, indicating that the Universidade de São Paulo, the Universidade Estadual de São Paulo, and the Universidade Estadual de Campinas are the academic institutions that receive the most financial support from Brazilian research funding agencies. 14

The Southern region secures the second position regarding the percentage of first affiliations mentioned in the publications analyzed. This finding mirrors the economic prosperity of the South – the second most affluent region in Brazil. Of note, two research support foundations from the Southern region (FAPERGS and FAPESC) appeared among the top 10 funding agencies. Moreover, data from the Ministry of Science, Technology, and Innovation (MCTI) 15 indicate that, in 2020, two states in the Southeast (São Paulo and Rio de Janeiro) and two in the Southern (Paraná and Santa Catarina) regions exhibited the highest proportions of financial expenditure on research and development relative to their total revenues – this directly contributes to the higher amount of research projects and publications from these regions.

More than 90% of the first, second, second-to-last and/or last authorships analyzed here were occupied by researchers affiliated with Brazil. This outcome implies that, despite more than ⅓ of the publications having international coauthorship, the authors without affiliations in Brazil predominantly occupied nonleading positions.

We also found relevant findings regarding female protagonism in dementia research. Compared to males, female protagonism was over 50% in all years, except between 2011 and 2012. Notably, we observed that female protagonism stands out at around 60% in the positions of first and second authors. In contrast, male protagonism is over 50% in the second-to-last and last author positions. Typically, the second-to-last and, mainly, the last authorships are occupied by senior researchers who often oversee the study's development; in contrast, junior researchers, such as Master's and Doctorate students, typically assume the positions of first and second authors. A potential explanation for the discussed results is that the higher proportion of men as second-to-last and last authors may reflect historical sex disparities, as women had fewer opportunities for academic positions in past decades. Consequently, the last two authorship positions encompass a larger number of male researchers who had more opportunities in the job market and academia. Conversely, a higher proportion of women as first and second authors reflects the more contemporary scenario, where women have been increasingly involved in academia and scientific research.

Of the 2,528 publications analyzed here, 1,812 (71.7%) were published in foreign and 716 (28.3%) in Brazilian journals. Notably, nearly half of the reports published in Brazil are concentrated in only two journals: Dementia e Neuropsychologia (31.4%) and Arquivos de Neuro-Psiquiatria (15.2%). The top five foreign journals are collectively responsible for 13.6% of studies on dementia published abroad – specifically, Journal of Alzheimer's Disease (5.6%), International Psychogeriatrics (2.4%), Current Alzheimer Research (2%), Plos ONE (1.9%), and International Journal of Geriatric Psychiatry (1.7%). These data underscore the notable difference in the number of foreign journals publishing on dementia compared to Brazilian journals. As mentioned earlier, the high prevalence of English language publications (95.1%) contributes to the internationalization of Brazilian research on dementia.

This bibliometric analysis showed that CNPq was the most cited research funder in the analyzed publications (19.5%), followed by CAPES (11.8%). Both were established in 1951 and are the most important research funding agencies in the country, playing a crucial role in the development and technological advancement in all 26 Brazilian states. Without the financial support and backing from these two agencies, the regional inequalities in scientific research, as demonstrated above, would certainly be much more pronounced.

In the wake of the last century's scientific development, São Paulo was a pioneer state, having created the first state research funding agency (FAPESP) in 1962. Over the subsequent two decades, this agency made substantial contributions to Brazilian scientific research by offering funding for doctoral scholarships abroad, particularly for researchers already employed by universities located in the State of São Paulo. This strategic initiative played a vital role in the internationalization of Brazilian research. 16

### Limitations

Bibliometric studies present limitations. 17 Here, we highlight the main drawbacks identified in our analyses. First, the literature search was confined to Scopus. Despite being a comprehensive and globally recognized database, however, it does not encompass all publications by authors affiliated with Brazil. Moreover, our literature search was limited to the period between 2010 and 2021, meaning that the results discussed here do not represent the scientific production from earlier decades.

In cases of conflict, we relied on the general objective of the study to classify each publication according to the research areas in dementia defined by the WHO. Nevertheless, there were instances where the publication under analysis could be classified in more than one category. For example, this occurred with literature reviews that summarized relevant data and information on various aspects and scenarios of dementia.

Furthermore, the analysis of the geographic regions linked to each publication was based on the first affiliation in Brazil of authors with leading positions in authorships, as we could not conduct a more comprehensive analysis covering all affiliations of all authors. For instance, there are publications with more than 15 authors and no automated tool is available to compute the respective geographic regions of Brazil linked to each publication.

We adopted a very narrow and basic approach to attribute sex to authors in leading positions, based on names and photos on Google Scholar, LinkedIn, ResearchGate, and other social networks. Hence, we must acknowledge the challenge of accurately determining sex solely through this method, which does not provide a comprehensive overview of gender differences. Moreover, the approach used here may not necessarily correspond to the authors' gender identity.

In conclusion, the current bibliometric review offers a comprehensive overview of dementia research in Brazil. Findings indicate that, between 2010 and 2021, the annual growth rate of publications on this theme closely paralleled that of general health-related literature, suggesting a foreseeable increase in dementia-related publications in the coming years.

A key takeaway from this review is the pressing need for additional studies across all areas of dementia research, especially in epidemiology and economic cost. Moreover, there is a call for the promotion and allocation of financial resources for dementia research in all regions of Brazil, to mitigate regional disparities in scientific output and enhance our understanding of the reality of dementia across all five geographic areas.
